# Population exposure to alcohol and junk food advertising during the 2018 FIFA world cup: implications for public health

**DOI:** 10.1186/s12889-022-13233-6

**Published:** 2022-05-06

**Authors:** Khaldoon Alfayad, Rachael L. Murray, John Britton, Alexander B. Barker

**Affiliations:** 1 UK Centre for Tobacco and Alcohol Studies, Division of Epidemiology and Public Health, Faculty of Medicine, University of Nottingham, Clinical Sciences Building, City Hospital, Nottingham, NG5 1PB UK; 2 Department of Public Health, College of Health Sciences, Saudi Electronic University, Riyadh Campus, Abi Bakr Siddiq Branch Rd, Riyadh, 13323 Saudi Arabia; 3 SPECTRUM Consortium, London, UK; 4 Psychology Department, Nottingham Trent University, 50 Shakespear Street, Nottingham, NG1 4FQ UK

**Keywords:** Alcohol, High fat sugar and salt, Impression, Children, Advertising, Exposure

## Abstract

**Background:**

Advertising alcoholic drinks and food high in fat, sugar, and salt (HFSS) is a driver of alcohol use and HFSS consumption, among children and young people. Whilst advertising legislation and broadcasting regulation protect children from alcohol and HFSS imagery, the 2018 FIFA World Cup, which attracted a global audience, was sponsored and partnered by alcohol and HFSS brands. This study investigated the exposure of viewers to HFSS and alcohol imagery in a selection of group matches, and the final match, of the FIFA 2018 World Cup.

**Methods:**

The frequency and duration of appearances (to the nearest second) of branding from two sponsors (McDonald’s and Budweiser), one official partner (Coca-Cola) and the official sports drink (Powerade) were recorded during all active play in live coverage of a sample of 13 matches (Six in Group A, which included the host nation, Russia, which has stringent alcohol promotion regulations in place; six in Group G, which featured England; and the final) broadcast in the UK. We used census and viewing data to calculate gross and per capita impressions generated by this imagery in the UK population.

**Results:**

The 13 matches included 1262 min of active play and a total of 1806 appearances of alcohol and HFSS food advertisements, delivering approximately 7.5 billion branded HFSS impressions, including 759 million to children (age < 16 years), and 3.3 billion alcohol impressions, including 385 million to children, in the UK. Appearances of HFSS and alcohol brands were not statistically different between the games in either group.

**Conclusion:**

UK advertising legislation and broadcasting regulations intended to prevent exposure to alcohol and HFSS imagery and advertising in UK television was circumvented completely by sponsorship arrangements in the 2018 FIFA World Cup. Preventing this exposure therefore requires revision of existing advertising and broadcasting controls to include sponsorship.

## Introduction

In 2018 alcohol consumption caused approximately 3 million deaths and around 5% of the total worldwide burden disability-adjusted life years (DALYs) lost to sickness and injury [1]. Exposure to alcohol marketing is associated with alcohol use and experimentation in young people [2, 3]. Alcohol product marketing, which includes promotion through sponsorship and other links to national and transnational sporting activities, is a thus a serious concern [4].

Consuming unhealthy food is also a major risk factor for non-communicable diseases including obesity, diabetes, cancers and cardiovascular disease [5]. There is a consensus that the current global obesity epidemic arises in large part from the increased availability and marketing of affordable, highly processed foods [6]. This is especially true of foods high in fat, sugar and/or salts (HFSS, or ‘junk food’) [7]. It has also been shown that exposure to HFSS food advertising (which includes sponsorship and other forms of promotion [8]) increases HFSS consumption among children [9–15] and adults [10, 13, 14].

Ofcom is the broadcast regulator in the UK [16], responsible for restricting representations of substances in children’s programming. The regulator also controls the glamorization of alcohol abuse in programming transmitted before 9 pm [17]. According to Section 1.10 of the Ofcom regulations, such content is likely to be accessed by should not be shown to children without editorial justification against public interest [18, 19]. Editorial justification refers to when the inclusion of certain content in a programme is justified with reference to the editorial requirements of a programme, for example where it is integral to the plot. Section 9.5 further states that ‘no excessive prominence may be granted to a product, service, or trademark in programming’ without an editorial reason. HFSS advertisement is often prohibited during or adjacent to programs commissioned by, primarily aimed at, or likely to cater to viewers under the age of 16 [18, 19]. Children under 16 years have a limited capacity to understand ads and are less likely to make responsible decisions in their consumption of HFSS foods [20]. However, Ofcom has no authority over sports sponsorship agreements, such as when a corporation sponsors a stadium, a team, or a single athlete, and Ofcom guidance notes that the context of advertising is taken into account, with more in situ advertising planned at sporting venues [21, 22]. This potentially represents a source of unregulated alcohol and HFSS advertising to children and young people.

The 2018 Fédération Internationale de Football Association (FIFA) World Cup finals, which appeal strongly to people of all ages, were sponsored or partnered by the alcohol brand Budweiser [23–25] and the HFSS brands, McDonald’s [23–25], Coca Cola [26] and Powerade [27]. The matches were held in Russia, a country with strict limitations on alcohol advertising [28]. We therefore present a content analysis of a selected sample of games from the 2018 FIFA World Cup to quantify the amount of imagery shown in a country with strict regulations and to estimate the subsequent population exposure to this imagery in the UK.

## Methods

The 2018 FIFA World Cup took place between June 14th and July 15th 2018 and involved a total of 63 matches, consisting of 48 group stage games, and 15 knockout games. To select games likely to attract some of the highest UK audiences we selected all matches from Group G (which included England) and the World Cup Final. We also included all Group A matches, as this group included Russia, the host nation, which has stringent alcohol promotion regulations in place and therefore may be expected to have a lower presence of alcohol imagery. Full details of the matches, date played and the UK terrestrial television channel which broadcast the match are given in Table 1. We measured all alcohol and HFSS advertising during all broadcast footage of active play in these matches from kick-off to the final whistle in the first and second halves of standard and extra time (none of the selected matches involved a penalty shootout). Our coding instrument separately listed each appearance of the HFSS brands ‘Coca Cola’, McDonalds’, ‘Powerade’, and the alcohol brand ‘Budweiser’ on digital advertising billboards along the perimeter of the pitch. For each appearance, start and end time in minutes and seconds (for example, 6:30 to 6:54) by match period (first and second half of normal, and stoppage time for each half) were recorded. Visual occurrences of each brand that appeared in clear, uninterrupted view on the screen received a single count in each instance. Information was recorded in separate Excel files for each match along with general information about the match (start time, end time, teams playing, date, broadcaster, stage in championship). To ensure the accuracy and reliability of coding, the TV coverage for three of the thirteen games was coded independently by two coders (KA and RM/AB) using the play, pause, review method previously reported [24, 25] and any differences resolved by discussion. UK viewing figures for the UK were supplied by Digital.I [29].

**Table 1:** Characteristics of the thirteen matches recorded

Parameters/Matches	Saudi vs Russia	Egypt vs Uruguay	Belgium vs Panama	England vs Tunisia	Russia vs Egypt	Saudi vs Uruguay	Belgium vs Tunisia	England vs Panama	Saudi vs Egypt	Russia vs Uruguay	England vs Belgium	Tunisia vs Panama	France vs Croatia
**Match date**	14/6/2018	15/6/2018	18/6/2018	18/6/2018	19/6/2018	20/6/2018	23/6/2018	24/6/2018	25/6/2018	25/6/2018	28/6/2018	28/6/2018	15/7/2018
**Kick off time**	16:00	13:00	14:00	19:00	19:00	16:00	13:00	13:00	15:00	15:00	19:00	19:00	16:00
**Tournament stage**	Group A	Group A	Group G	Group G	Group A	Group A	Group G	Group G	Group A	Group A	Group G	Group G	Final
**Channel**	ITV	BBC	BBC	BBC	BBC	BBC	BBC	BBC	ITV	ITV	ITV	ITV	BBC/ITV
**Active playing time (sec)**	5801	5832	5769	5830	5652	5759	5832	5842	6129	5808	5588	5977	5904
**% viewing (under 16 years)**	3.9%	0.9%	3.2%	17.3%	6.4%	3.3%	3.75	15.7%	0.17%	1.2%	15.9%	0.02%	9.3%
**% viewing (16 years and over)**	7.0%	4.6%	7.3%	30%	12.6%	6.1%	7.2%	22.4%	0.31%	4.5%	23.9%	0.1%	15.2%

To estimate UK population exposure to branding content we analysed the distribution of branding appearances and used that distribution to compute cumulative gross and per capita impressions, using previously reported methods [30, 31]. To generate the cumulative distributions of branding appearances by match and type of visual occurrence (McDonalds, Powerade, Budweiser and Coca Cola) we disaggregated the data on total duration of each visual occurrence to second-by-second observations by match period.

Viewership was calculated from proportion viewership figures from *Digital.i* (http://www.digital-i.com/) and UK mid- year population estimates in 2018 from census data [32]. Viewership was then combined with the number of alcohol and HFSS appearances per match to provide gross impressions, and gross impressions divided by population estimates to provide per capita impressions for children (4 to 15 years old) and total (less than16 years and above) in the UK.

## Results

Seven matches were broadcast in the UK by the British Broadcasting Corporation (BBC) and six by the Independent Television Network (ITV). The total duration of active play for the 13 matches was 75,731 s (1,262 min and 11 s). Games were viewed by between 0.1 and 30% of the UK adult and 0.02 to 17.3% of UK child population (Table 1).

We identified 1806 instances of brand appearance in the sampled broadcasts, comprising 602 (33.3%) for McDonald’s, 551 (30.4%) for Budweiser, 464 (25.7%) for Coca-Cola, and 189 (10.5%) for Powerade (Table 2).

**Table 2:** Match by match analysis of adverts for McDonald’s, Coca-Cola, Powerade, and Budweiser

Parameters	Saudi vs Russia	Egypt vs Uruguay	Belgium vs Panama	England vs Tunisia	Russia vs Egypt	Saudi vs Uruguay	Belgium vs Tunisia	England vs Panama	Saudi vs Egypt	Russia vs Uruguay	England vs Belgium	Tunisia vs Panama	France vs Croatia	Total
Number of McDonald’s brand appearances	58	50	59	54	40	36	47	40	40	41	33	53	51	602
Number of Coca Cola brand appearances	44	29	35	41	31	39	34	27	31	30	29	46	49	464
Number of Budweiser brand appearances	62	39	46	41	37	35	35	30	47	40	38	49	52	551
Number of Powerade brand appearances	11	12	12	19	15	12	13	14	16	15	16	17	17	189
Total number of brand appearances	175	130	152	155	123	122	129	111	134	126	116	165	169	1806
Duration of McDonald’s brand appearances	404	441	409	465	370	305	492	463	445	304	416	379	373	5266
Duration of Coca Cola brand appearance	367	335	364	367	298	290	299	371	316	270	323	286	389	4275
Duration of Budweiser brand appearances	428	389	401	461	345	414	407	403	483	319	416	390	369	5225
Duration of Powerade brand appearances	203	177	106	192	174	175	177	180	166	195	166	169	163	2243
Total duration of brand appearances	1402	1342	1280	1485	1187	1184	1375	1417	1410	1088	1321	1224	1294	17,009
% of playing time where brand appearance occurs	24.1%	23.0%	22.2%	25.5%	21.0%	20.5%	23.6%	24.2%	23.0%	18.7%	23.6%	20.5%	21.9%	22.5%

There was variation across the numbers of brand appearances in games played by Russia compared to the other matches involving other countries in the same group. Brand appearances for Russia matches were non-statistically significantly higher (424 appearances) compared to other Group A matches involving other countries (386 appearances) (Table 2). This comprises of 139 alcohol appearances in games played by Russia compared to 121 alcohol appearances in matches played by other Group A teams. Similarly, HFSS appearances in matches played by Russia (285 appearances) is slightly higher than HFSS appearance in matches played by other Group A teams (265 appearances) (Table 2). There was also variation across the matches played by England compared to the other Group G matches. However, brand appearance in matches played by England (382, comprising of 109 alcohol and 273 HFSS brands) is lower compared to brand appearance on other Group G matches (446, comprising of 130 alcohol and 316 HFSS brands) (Table 2). The occurrence of brand appearances varied significantly across the 13 games: being highest in the game between Russia and Saudi Arabia (175 appearances), and lowest in the England - Panama game (111 appearances) (Table 2).

The total duration of brand appearances across the 13 matches was 17,009 s (283 min 5 s) or 22.5% of all playing time), of which the McDonald’s brand appeared for 5266 s (7.0% of playing time), Budweiser for 5225 s (6.9% of playing time), Coca Cola for 4275 s (5.6% of total playing time) and Powerade for 2243 s (3.0% of total playing time, Table 2). The frequency of duration of brand appearances varied across the 13 games: being highest in the game between England and Tunisia (1485 s), and lowest in the game between Russia and Uruguay (1108 s) (Table 2).

Nearly half of all brand appearances (840 appearances, 46.5%, lasting 85 min 48 s) occurred on billboards along the side-lines of the pitch; 232 appearances (12.8%: 18 min, 5 s) were on billboards behind the goal lines (Table 3), and 734 appearances (40.6%: 179 min, 18 s) occurred simultaneously on side-line and goal-line billboards.

**Table 3:** Location, frequency, and duration (seconds) of brand appearance during the thirteen selected matches of the 2018 FIFA World Cup

Pitch side	MacDonald’s	Coca Cola	Powerade	Budweiser	Total
Total number of sideline appearances	319	239	31	251	840
Total number of goal line appearances	69	59	14	90	232
Total number of simultaneous side lines and goal line appearances	214	166	144	210	734
Overall total number of appearances % of total	602 33.3%	464 25.7%	189 10.5%	551 30.5%	1806 (100%)
Total duration of sideline appearances	1892 s	1319 s	194 s	1743 s	5148 s
Total duration of goal line appearances	371 s	281 s	55 s	403 s	1110 s
Total duration of simultaneous side lines and goal line appearances	3003 s	2675 s	1994 s	3079 s	10,751 s
Overall total duration of appearances % of total	5266 s (30.9%)	4275 s (25.1%)	2243 (13.2%)	5225 s (30.7%)	17,009 s (100%)

**Fig. 1:**
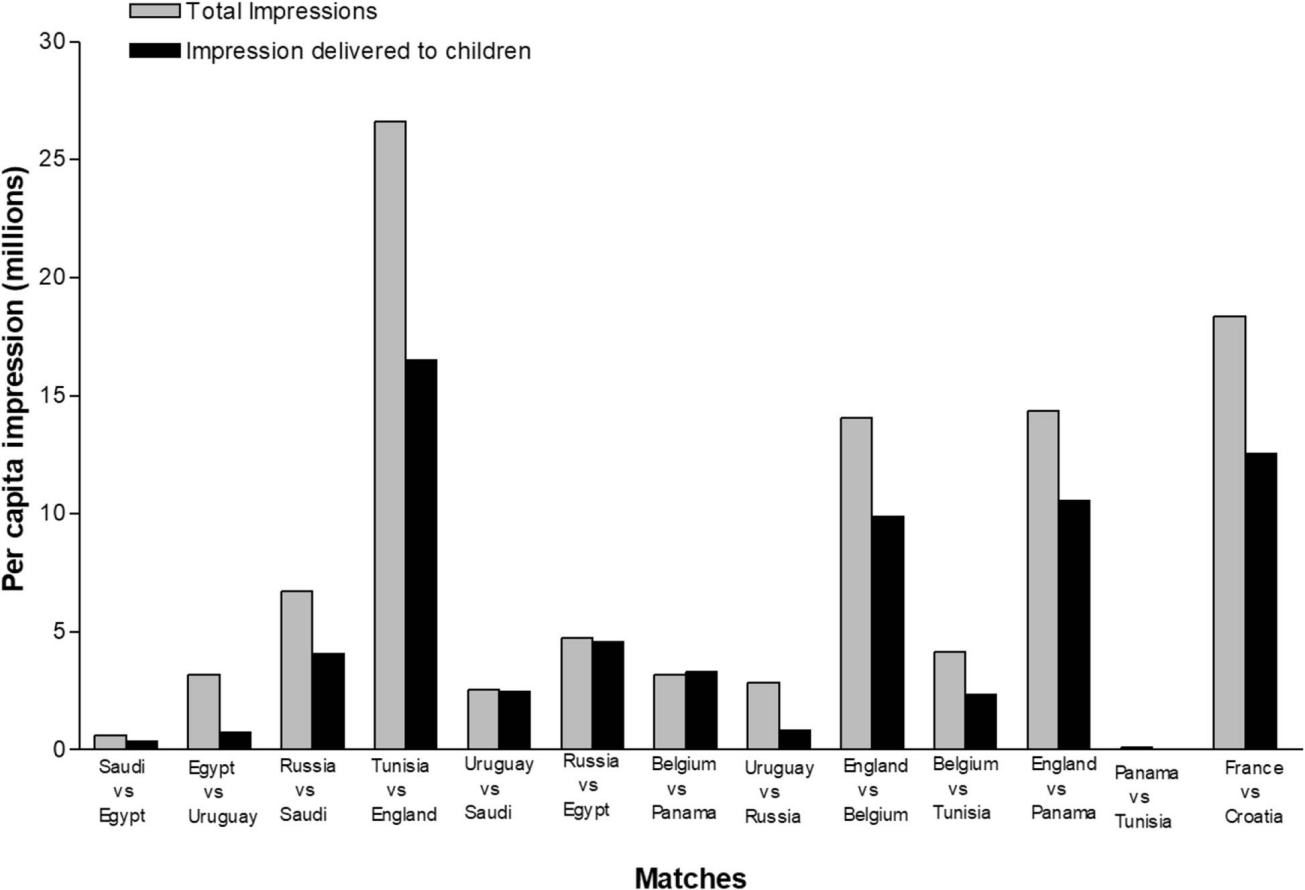
Per capita total impression, and impressions delivered to children of HFSS in selected matches during the FIFA 2018 World Cup

**Fig. 2:**
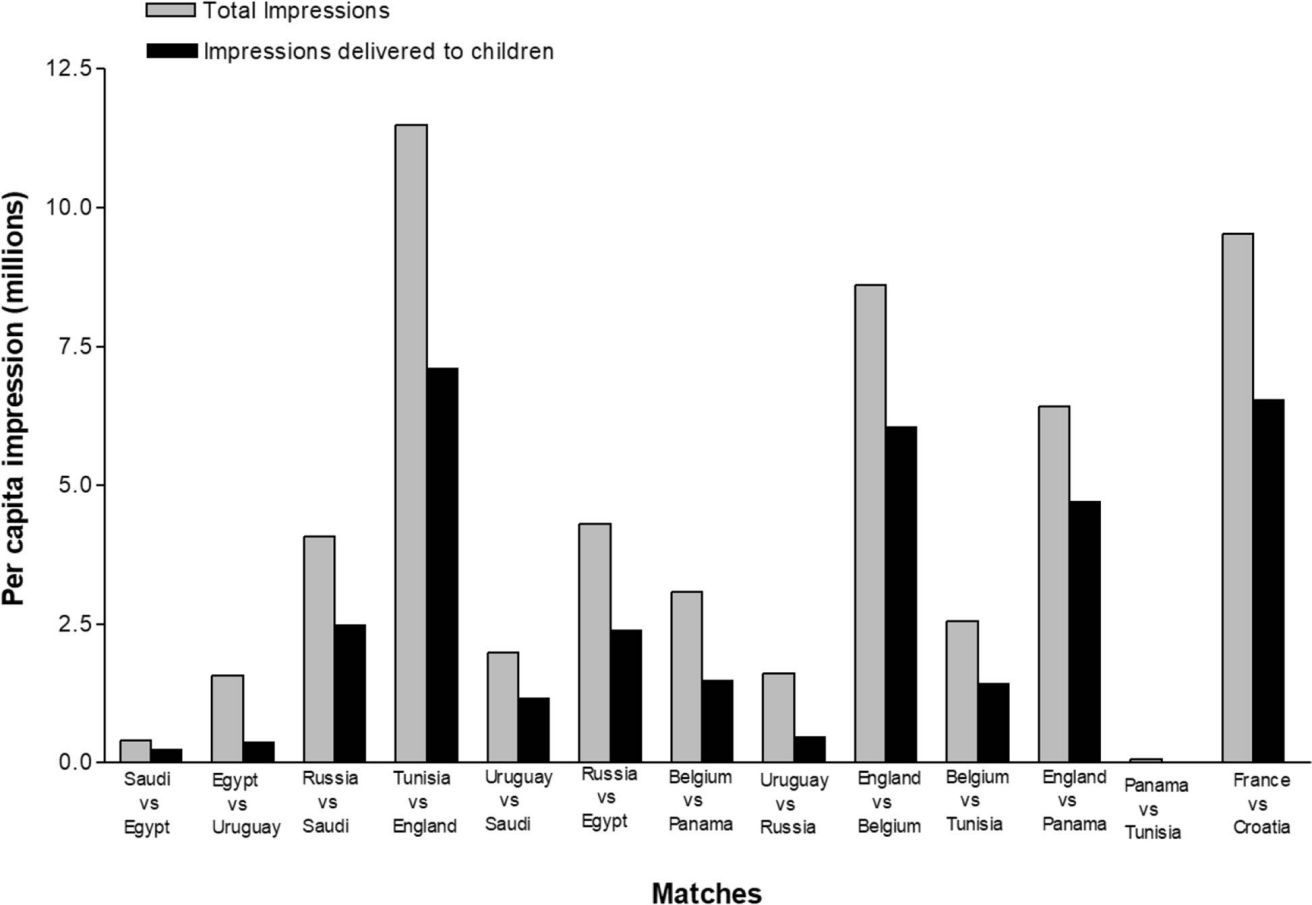
Per capita total impression, and impression delivered to children of alcohol in selected matches during the FIFA 2018 World Cup

In total, the 13 games delivered an estimated 6.7 billion gross branded HFSS impressions and 3.7 billion gross branded alcohol impressions to UK viewers (Table 4). Estimated total HFSS food and alcohol impressions delivered to viewers varied significantly across the selected matches, with Tunisia and England’s match showing the highest numbers for HFSS (1.8 billion), and alcohol (763.2 million). While on the other hand, the lowest numbers recorded were observed in Panama and Tunisia’s match, (with HFSS at 7.9 million and alcohol at 3.9 million) (Table 4). Per capita HFSS impressions delivered by the sample matches are shown in Fig. 1. The analysis of per capita impressions for alcohol, indicating a similar pattern, is presented in Fig. 2.

**Table 4:** Gross and per capita total impressions of cumulative HFSS and alcohol appearances by population group and match

Match	HFSS		Alcohol	
	Gross Impression (95% CI) (Billion)	Per capita Impression (95% CI) (Billion)	Gross Impression (95% CI) (Billion)	Per capita impression (95% CI) (Billion)
Saudi v Egypt	41.04 (27.62–54.46)	0.61 (0.42–0.82)	27.38 (18.53–36.22)	0.41 (0.28–0.55)
Egypt v Uruguay	210.58 (192.43–228.75)	3.17 (2.90–3.45)	104.02 (95.15–112.88)	1.57 (1.43–1.70)
Russia v Saudi	446.26 (416.63–475.91)	6.72 (6.27–7.17)	271.41 (253.59–289.22)	4.09 (3.82–4.35)
Tunisia v England	1767.32 (1717.35–1817.30)	26.61 (25.86–27.37)	763.15 (741.82–784.47)	11.49 (11.17–11.81)
Uruguay v Saudi	171.27 (155.27–187.29)	2.57 (2.34–2.82)	131.61 (122.25–140.98)	1.98 (1.84–2.12)
Russia v Egypt	316.71 (295.90–337.53)	4.76 (4.46–5.08)	286.07 (272.33–299.81)	4.31 (4.10–4.51)
Belgium v Panama	211.73 (191.80–231.67)	3.18 (2.89–3.49)	205.30 (191.97–218.64)	3.09 (2.89–3.29)
Uruguay v Russia	188.58 (172.28–204.88)	2.84 (2.59–3.09)	106.30 (97.22–115.38)	1.60 (1.46–1.74)
England v Belgium	932.73 (902.34–963.14)	14.04 (13.59–14.51)	571.98 (553.56–590.41)	8.61 (8.33–8.89)
Belgium v Tunisia	276.06 (257.90–294.23)	4.15 (3.88–4.43)	169.29 (158.28–180.30)	2.55 (2.38–2.71)
England v Panama	952.67 (920.49–984.87)	14.34 (13.86–14.83)	426.80 (412.55–441.06)	6.42 (6.21–6.64)
Panama v Tunisia	7.89 (3.88–11.91)	0.11 (0.06–0.18)	3.91 (1.94–5.88)	0.06 (0.03–0.09)
Final Total (BBC + ITV)	1187.23 (1126.58–1247.88)	18.34 (17.39–19.29)	617.36 (585.82–648.90)	9.53 (9.04–10.03)

Gross impression delivered to children across the 13 coded matches were recorded separately (Table 5). Branded HFSS impression delivered to children ranged between 220, 000 (observed for the match between Panama and Tunisia) and 208 million (observed for the match between Tunisia and England) while the sum of branded HFSS impressions delivered to children was 852 million (Fig. 1). Alcohol impressions delivered to children ranged from 110, 000 (Panama v Tunisia) to 89.6 million (Tunisia v England), a total of 354 million impressions (Fig. 2).

**Table 5:** Gross and per capita child impressions of cumulative HFSS and alcohol appearances by population group and match

Match	HFSS		Alcohol	
	Gross Impression (95% CI)	Per capita Impression (95% CI)	Gross Impression (95% CI)	Per capita impression (95% CI)
Saudi v Egypt	4.49 (2.56–6.43)	0.35 (0.20–0.51)	2.99 (1.71–4.27)	0.24 (0.14–0.34)
Egypt v Uruguay	9.31 (7.63–11.01)	0.73 (0.61–0.87)	4.59 (3.77–5.42)	0.36 (0.30–0.43)
Russia v Saudi	51.56 (47.12–56.02)	4.09 (3.74–4.45)	31.30 (28.63–33.97)	2.48 (2.27–2.69)
Tunisia v England	207.91 (199.91–215.91)	16.50 (15.87–17.14)	89.60 (86.19–93.02)	7.10 (6.83–7.37)
Uruguay v Saudi	31.43 (28.45–34.43)	2.49 (2.26–2.73)	14.65 (13.27–16.03)	1.16 (1.05–1.27)
Russia v Egypt	57.65 (53.78–61.53)	4.57 (4.27–4.88)	30.01 (28.01–32.00)	2.38 (2.22–2.53)
Belgium v Panama	38.19 (34.51–41.89)	3.03 (2.74–3.32	18.72 (16.94–20.51)	1.48 (1.34–1.62)
Uruguay v Russia	10.37 (8.69–12.06)	0.82 (0.69–0.96)	5.84 (4.90–6.78)	0.46 (0.39–0.54)
England v Belgium	124.54 (119.50–129.59)	9.88 (9.48–10.29)	76.23 (73.17–79.28)	6.04 (5.80–6.28)
Belgium v Tunisia	29.51 (26.89–32.14)	2.34 (2.13–2.55)	18.06 (16.47–19.66)	1.43 (1.30–1.56)
England v Panama	133.05 (127.63–138.48)	10.55 (10.13–10.99)	59.49 (57.09–61.89)	4.71 (4.52–4.90)
Panama v Tunisia	0.22 (− 0.07–0.53)	0.01(−0.01–0.04)	0.11 (− 0.03–0.26)	0.01 (0.00–0.02)
Final Total (BBC + ITV)	154.00 (144.15–163.85)	12.56 (11.75–13.38)	80.01 (74.95–85.20)	6.53 (6.11–6.95)

The study compared brand appearances in the matches played in Groups A and G. The findings revealed a similar rate of brand appearance between the two groups (Table 6). Despite the similarities, HFSS brand appearances were slightly higher in Group G matches (589) compared to Group A matches (550). Group A had more alcohol brand appearances (260) compared to Group G (239). The duration of brand appearance was higher in Group G (8120 s) compared to Group A (7613 s). Further analysis showed that the duration for HFSS imagery (5642 s) and alcohol (2478 s) in Group G was higher compared to (5235 s) for HFSS and (2378 s) for alcohol in Group A (Table 6).

**Table 6:** Duration, appearances, and comparison between Group A and G

Description	Group A	Group G	***P*** Value
HFSS appearance	550	589	0.435
Alcohol appearance	260	239	0.499
**Total appearance for the group**	**810**	**828**	**0.812**
Duration of active play	34,985 s (583 min 8 s)	34,838 s (581 min 3 s)	0.781
HFSS duration	5235 s (87 min, 25 s)	5642 s (94 min)	0.213
Alcohol duration	2378 (40 min, 3 s)	2478 s (41 min, 3 s)	0.539
**Total Duration of brand**	7613 s	8120 s	0.252

## Discussion

This study identified 1806 brand appearances during the 13 matches selected for analysis. The McDonald’s brand was the most commonly observed (33.3%) followed by Budweiser (30.5%), Coca-Cola (25.7%) and Powerade (10.5%). HFSS brand appearance accounted for about 69.5% (McDonald’s, Coca-Cola, and Powerade) of all brand appearances during the selected matches, while alcohol brand appearance accounted for the remaining 30.5% (Budweiser). Brand appearance (both HFSS and alcohol) across Group G matches was higher than group A matches, and higher in matches played by the host country (Russia, despite the prohibition of alcohol advertisement) than other group G matches, though these difference are not statistically significant. The duration of brand appearance accounted for 22.5% of the total playing time for the 13 selected matches and the appearance of McDonald’s, Budweiser, Coca Cola, and Powerade accounted for 7, 6.9, 5.6, and 3% of playing time respectively. The majority of brand appearance (46.5%) occurred along pitch side-lines alone while 40.6% of all brand appearance occurred simultaneously on the side-line and goal line. These brand appearances generated substantial audience exposure, delivering 3.7 billion of alcohol and 6.7 billion of HFSS total gross impressions. This study also revealed that 852 million HFSS and 354 million alcohol impressions were also delivered to UK children who watched the 13 selected matches. Our study thus provides evidence that the 2018 FIFA World Cup was a source of significant exposure of children, young people, and adults to branded HFSS and alcohol advertising through sports sponsorship and is likely to be a contributor to alcohol and HFSS consumption by young people and adults.

Available evidence indicates that advertising of alcohol and HFSS, particularly among children, can influence eating behaviour [33, 34] and food choices [14], leading to an increased risk of obesity and related morbidities [35, 36]. Advertising during sporting events is a common practice and has been identified as the dominant medium for the promotion of alcohol and drinking among the general population [34]. Budweiser and Coca Cola have historically been major sponsors of several sporting events, such as stock car racing, the Olympics and major football competitions [37]. Budweiser and Coca Cola spent $350 million and $265 million respectively for sports sponsorship in 2016 [37]. McDonald’s has been top sponsor of the Olympics and contributed around $1 billion every four years before ending the sponsorship in 2018 [38]. Powerade is also an official sponsor of many international sport events, including Rio 2016 Olympic Games, Australian Olympic Committee, football events, rugby union, and cricket [27].

Though the advertising of alcohol and HFSS to adults is allowed in the UK, such advertisements are subject to regulations intended to protect children and young adults, particularly when the percentage of young viewers exceeds 30% of the target audience [39]. With respect to alcohol, the code seeks to prevent the general appeal of these products to children and young adults [18, 19, 39]. However, while the Ofcom broadcasting code restricts content in programmes, the regulator has no remit over sponsorship at televised sporting events and the Advertising Standards Authority, the UK’s regulator of advertising, does not regulate advertisements at the venue of televised sporting events due to their definition of advertising [1]. Alcohol and HFSS advertising through sponsorship at televised sporting events is thus, to practical purposes, currently unregulated.

Our analysis shows that the 2018 FIFA world cup was a major source of exposure to children and young people in the UK and is likely to be a contributor to HFSS consumption and alcohol use. These results are in accordance with findings reporting that advertising of alcohol, particularly among children, can influence behaviour [33], leading to an increase in the risk of related morbidities [35]. The earlier children are exposed to alcohol advertising, the earlier they start drinking [40, 41]. Children who otherwise might not have been thinking about alcohol start thinking to themselves ‘is this the product for me’ whenever they see alcohol advertisements [40, 41]. If these young people are already drinking, exposure to alcoholic content increases their chances of drinking at hazardous levels [40]. Despite EU regulations which prohibit media advertisement of HFSS and alcohol related contents to children, pitch-side promotional appearances during active play of the FIFA 2018 World Cup totalled over 2.5 h for HFSS brands and approximately 1.5 h for alcohol brands. Given the potential influence of this exposure on food choices and alcohol consumption among children and young adults, it is important that current regulations include televised sporting events in their remit to prevent young people being exposed to this content. In France, the “Loi Evin” otherwise referred to as the Evin’s Law largely controls alcohol marketing and bans alcohol advertising. However, Big Alcohol keeps breaking the law or tries to circumvent it despite the legal repercussions [42]. It is also imperative that global advertisement strategies which ensure benefits to sporting event sponsors without jeopardizing the health and well-being of the population are developed for the future.

Our findings lend support to studies calling for comprehensive regulation of food (and beverage) advertising during peak viewing hours accessible to children. Similar to our study, Kelly et al. recommend that regulation of TV advertising aimed at children should concentrate on the type of programs where advertisements are broadcast, the type of product, the target audience, the time of day, and the subject matter of advertisements [43]. At the same time, regulators must also consider focusing on the addition of sponsorship and sport to the scope of comprehensive regulations. Current self-regulatory marketing codes targeting alcohol and food are ineffective since most ignore the sponsorship of sport.

The cross-sectional nature of our study means that we are unable to estimate the effect of the documented exposure on HFSS or alcohol content consumption in our study population. However, there is evidence from elsewhere that exposure to such imagery through other media increases consumption of alcohol and HFSS [44]. We only coded a sample of 13 of the 48 matches in the entire FIFA World Cup competition. However, we have no reasons to suspect that the other groups and games would have been different, given our finding of the similarity of alcohol appearances in games featuring countries with different controls on alcohol advertising in place.

The 13 games delivered an estimated 6.7 billion gross branded HFSS impressions and 3.7 billion gross branded alcohol impressions to UK viewers. Our estimation of both gross and per capita impressions in this study assumes that viewers watched the entire broadcast of matches selected for coding and analysis, when in fact many may have watched only parts of the games. Calculating the gross and per capita impressions to measure population exposure has certain implications. The alcohol industry frequently cites gross impressions as a more suitable means to measure alcohol advertising [45]. However, the disparity in population size causes more impressions per person for youth and fewer per person for adults. Moreover, we also only coded a small proportion of the matches featured in the 2018 World Cup (21% of matches) and this indicates that exposure arising from the full competition is likely to be substantially higher. Also, the study is unable to capture impressions to viewers who watched selected matches online, from within the stadia or viewers of other matches played throughout the tournament. For this reason, figures we have provided are likely to underestimate true exposure. The British Broadcasting Corporation (BBC) indicated that 44.5 million people watched its coverage of the FIFA World Cup on television while a further 49.2 million people watched online via the BBC Sport website [46]. England played in some of the matches coded in this study and this may partly account for higher viewing figures for those matches, compared to matches involving other countries. The global viewership of the FIFA World Cup has been estimated at 3.4 billion, which is nearly half the global population [47]. This includes home TV audiences (estimated at 160 million), those who watched the game online and others who watched in public places such as bars, outdoor locations and pubs [46]. The UK exposure figures therefore probably represent a very small proportion of the true total global exposure.

## Conclusion

This study has thus demonstrated that the 2018 FIFA World Cup was a source of significant exposure of branded HFSS and alcohol advertising through sports sponsorship and is likely to be a contributor to alcohol and HFSS consumption by young people. Future studies should continue to monitor alcohol and HFSS advertising through sponsorship at sporting events, to explore the population exposure to unregulated HFSS and alcohol advertising and policies reviewed to include restrictions on sports sponsorship to reduce exposure to alcohol and HFSS advertising through this medium.

## Data Availability

The datasets used and/or analysed during the current study are available from the corresponding author on reasonable request.
